# Developing bones are differentially affected by compromised skeletal muscle formation

**DOI:** 10.1016/j.bone.2009.11.026

**Published:** 2010-05

**Authors:** Niamh C. Nowlan, Céline Bourdon, Gérard Dumas, Shahragim Tajbakhsh, Patrick J. Prendergast, Paula Murphy

**Affiliations:** a Department of Zoology, School of Natural Sciences, Trinity College Dublin, Ireland; b Trinity Centre for Bioengineering, School of Engineering, Trinity College Dublin, Ireland; c Stem Cells & Development, Department of Developmental Biology, Institut Pasteur, Paris, France

**Keywords:** Mechanobiology, Mechanical forces, Embryonic bone formation, Muscle contractions, Endochondral ossification

## Abstract

Mechanical forces are essential for normal adult bone function and repair, but the impact of prenatal muscle contractions on bone development remains to be explored in depth in mammalian model systems. In this study, we analyze skeletogenesis in two ‘muscleless’ mouse mutant models in which the formation of skeletal muscle development is disrupted; *Myf5^nlacZ/nlacZ^:MyoD^−/−^* and *Pax3*^*Sp/Sp*^ (*Splotch*). Ossification centers were found to be differentially affected in the muscleless limbs, with significant decreases in bone formation in the scapula, humerus, ulna and femur, but not in the tibia. In the scapula and humerus, the morphologies of ossification centers were abnormal in muscleless limbs. Histology of the humerus revealed a decreased extent of the hypertrophic zone in mutant limbs but no change in the shape of this region. The elbow joint was also found to be clearly affected with a dramatic reduction in the joint line, while no abnormalities were evident in the knee. The humeral deltoid tuberosity was significantly reduced in size in the *Myf5^nlacZ/nlacZ^:MyoD^−/−^* mutants while a change in shape but not in size was found in the humeral tuberosities of the *Pax3*^*Sp/Sp*^ mutants. We also examined skeletal development in a ‘reduced muscle’ model, the *Myf5^nlacZ/+^:MyoD^−/−^* mutant, in which skeletal muscle forms but with reduced muscle mass. The reduced muscle phenotype appeared to have an intermediate effect on skeletal development, with reduced bone formation in the scapula and humerus compared to controls, but not in other rudiments. In summary, we have demonstrated that skeletal development is differentially affected by the lack of skeletal muscle, with certain rudiments and joints being more severely affected than others. These findings indicate that the response of skeletal progenitor cells to biophysical stimuli may depend upon their location in the embryonic limb, implying a complex interaction between mechanical forces and location-specific regulatory factors affecting bone and joint development.

## Introduction

Mechanical forces are known to be essential for adult bone maintenance and repair [1,2], and it is thought that the mechanical environment in the developing limb has an important influence on embryonic bone and joint formation [3–6]. For instance, it has been shown that infants with neuromuscular diseases, which cause reduced movement *in utero*, have long bones which are thin, hypo-mineralized and prone to fractures [3]. In children affected with hemiplegic cerebral palsy, skeletal maturation in the affected side is delayed [7]. An enhanced understanding of the mechanics of bone and joint formation during development will provide vital clues to the mechanoregulation of cells and tissues, and could potentially lead to better treatments for conditions where skeletal development is affected by reduced movement *in utero*. Studying the relationship between mechanical forces and skeletal development can contribute to skeletal tissue engineering, where aspects of developmental processes are emulated *in vitro* [8].

Mouse mutants in which muscle development is affected provide useful systems for examining skeletogenesis in the presence of altered mechanical environments. Two such strains are *Myf5^nlacZ/nlacZ^:Myod^−/−^* [9] and *Pax3*^*Sp/Sp*^ (*Splotch*) [10]. *Pax3*^*Sp/Sp*^ mutants lack the transcription factor *Pax3*, which is critical for the migration of muscle stem/progenitor cells into the limb buds [11], therefore these mutants lack limb muscles. Pax3 is required in multiple developing systems and mutations also cause neural tube [12] and cardiac defects [13], which can lead to the death of homozygous mutants *in utero* from embryonic day E14 [14]. Abnormalities of the skull, ribs and vertebrae, and fusion of bones in the shoulder and hip regions have also been noted in *Pax3* null mouse embryos [14]. In humans, heterozygous mutations of the gene cause Waardenburg syndrome, with symptoms including hearing loss and pigmentation abnormalities [15]. In *Myf5^nlacZ/nlacZ^:Myod^−/−^* double mutants, the function of three myogenic determination genes is abrogated: *Myf5*, *Myod*, and *Mrf4* (*Mrf4* function compromised *in cis*) [9]. Muscle stem/progenitor cells migrate into the limbs of mutants, but they do not commit to the myogenic fate, and consequently myoblasts and differentiated muscle fibers are lacking [9,16]. Rot-Nikcevic et al. [17,18] examined skeletogenesis in the absence of skeletal muscle in late stage (E18.5) *Myf5^−/−^:Myod^−/−^* fetuses, a comparable model with a different knockout allele of *Myf5*. Features of the immobilized embryos included a shorter scapula, clavicle, mandible, femur and tibia, an abnormal sternum and absence of the humeral deltoid tuberosity, with unchanged lengths of the humerus, radius or ulna [17]. The muscleless mice had reduced separation between the radius and ulna, and between the tibia and fibula [17]. Gomez et al. [19] also reported absence of the humeral tuberosity in the *Myf5^−/−^:Myod^−/−^* model, but in contrast to the Rot-Nikcevic study [17], reported significantly shorter ulnae at E18 [19]. Gomez et al. [19] found thicker humeri and femora in the E18 mutants, with an increased cortical thickness in the femur. An increase in the number of osteoclasts in the tibia and fibula was found, but histology revealed no differences in ossification between the phalanges or femora of mutant and wildtype animals at E18 [19].

While there is evidence to suggest that the absence of muscle can affect the shape and size of different skeletal elements, there are limited data on how the initiation and maintenance of ossification may be affected by an altered mechanical environment in the mammalian limb. However, data from chick immobilization studies suggest that mechanical forces due to muscle contractions may play an important role in bone initiation and maintenance. Hosseini and Hogg [5] examined the timing and extent of ossification centers in immobilized chick limbs, and noted that, while the timing of the initial appearance of ossification centers was similar in immobilized and control limbs, by 19 days there was a reduction of between 25% and 33% in the length of the calcified diaphysis in the major long bones. Studies using the chick have investigated possible mechanisms underlying the mechanoregulation of embryonic bone. Germiller and Goldstein [20] observed a decrease in proliferation of chondrocytes in the avian embryonic growth plate as a result of immobilization, and proposed that skeletal muscle contractions play a role in the regulation of immature chondrocytes. We showed previously that the ossification of the avian tibia was affected by an altered mechanical environment and proposed, based on changes in gene expression in immobilized limbs, that ColX and Ihh may play a role in mechanoregulatory pathways contributing to bone formation [21].

In this study, we characterize bone development in forelimb and hindlimb skeletal elements in two ‘muscleless’ mouse mutant strains; *Myf5^nlacZ/nlacZ^:Myod^−/−^*, and *Pax3*^*Sp/Sp*^ with particular emphasis on the extent of early ossification. As both mutants are completely devoid of skeletal muscle in the limbs, the skeletal elements develop in the absence of neighboring muscle tissue, and therefore lack dynamic patterns of biophysical stimuli that we have shown to result from spontaneous embryonic muscle contractions [22]. In littermates of the double knockout, when one functional copy of *Myf5* is present (*Myf5^nlacZ/+^:Myod^−/−^*), skeletal muscle differentiation occurs, but the number of muscle fibers, and muscle mass, is decreased by 35–55% [23]. We also examine skeletogenesis in these ‘reduced muscle’ *Myf5^nlacZ/+^:MyoD^−/−^* embryos. We test the hypothesis that the initiation and progression of ossification are affected by the lack of skeletal muscle by examining two independent genetic lesions leading to muscle absence, and we identify the skeletal elements which are most affected by the altered mechanical environment in the developing limb.

## Methods

### Animal models and generation of embryonic samples

*Myf5^nlacZ/+^:Myod^+/−^* or *Pax3*^*Sp/+*^ were interbred either by spontaneous matings or by superovulating females and offspring were subsequently genotyped as described previously [9,10]. Embryos and fetuses were harvested at E14.5, E14.75 and E15.5 and each embryo was staged using Theiler morphological criteria [24] with particular focus on anatomical features likely to be unaffected by the lack of muscle, such as external features of skin, eye and ear development, in conjunction with limb features such as separation of the digits. For example, embryos were designated Theiler stage (TS) 23 if the fingers and toes were separated and divergent, the eyelids open, the skin smooth and the pinna of the ear not yet fully covering the ear canal [24]. Ten *Myf5^nlacZ/nlacZ^:Myod^−/−^* and eleven *Myf5^nlacZ/+^:MyoD^−/−^* embryos at stage TS23 were analyzed and compared with thirteen littermates with normal skeletal muscle at TS23, used as controls for these groups. The control genotypes included; *Wt, Myf5^nlacZ/+^:Myod^+/-^, Myf5^nlacZ/+^:Myod^+/+^* and *Myf5^+/+^:Myod^-/+^*. *Pax3^Sp/Sp^* embryos at TS23 (*n* = 7) and TS25 (*n* = 3) were compared with stage-matched control littermates (*n* = 9 at TS23 and *n* = 6 at TS25). TS23 is the stage at which primary centers of ossification initiate in the major long bone rudiments [25], while at TS25, bone centers are established and progressing in the major long bone rudiments [25].

### Morphological analysis

**Fig. 1:**
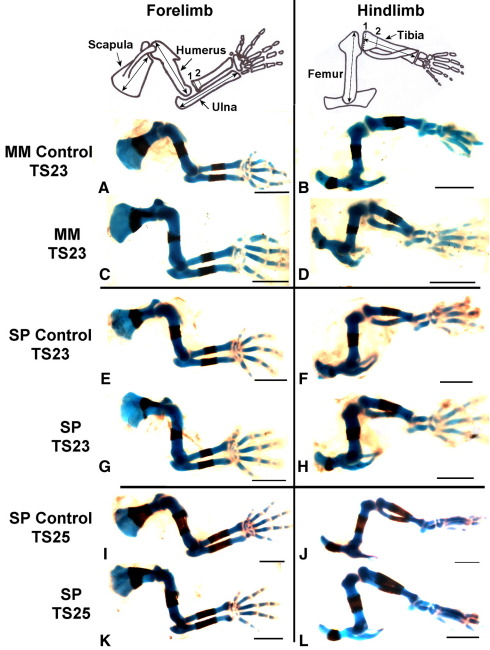
Gross morphology of forelimbs and hindlimbs from mutant groups *Myf5^nlacZ/nlacZ^:Myod^−/−^* (MM) and *Pax3*^*Sp/Sp*^ (SP) with stage-matched control littermates at TS23 and TS25 (SP only). Double ended arrows on the anatomical drawings show where rudiment length measurements were taken. Dotted (numbered) lines on the radius and tibia show where width measurements were taken from cross sections of 3D reconstructions of OPT scanned limbs. Scale bars 1 mm.

**Fig. 2:**
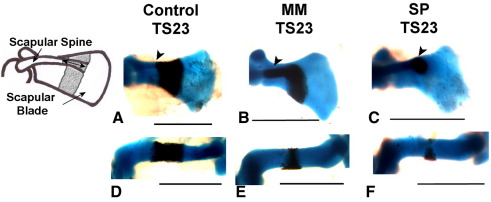
Scapular and humeral abnormalities in limbs devoid of skeletal muscle in *Myf5^nlacZ/nlacZ^:Myod^−/−^* (MM) and *Pax3*^*Sp/Sp*^ (SP) mutants. In control embryos, ossification of the scapular spine (arrows) and scapular blade progress to the same degree (A), while in mutants, ossification of the scapular spine extends beyond ossification of the blade, as shown in panels B and C. Scapular bone was often found to be incomplete across the width of the scapular blade as shown in panel B, or absent from the blade as shown in panel C (circular region is calcified base of scapular spine). The territory of forming humeral bone was found to be irregular in mutants, with more on the posterior aspect of the rudiment (E, F), compared to the regular bone collar seen in the controls (D). Scale bars 1 mm.

The embryos were divided into two halves along the midline of the anterior–posterior axis, with one half stained for cartilage and bone using Alcian blue and Alizarin red as detailed in Hogan et al. [26]. The stained specimens were photographed, and the forelimbs and hindlimbs were then removed and photographed individually in a consistent manner. Any abnormal features of the skeletal rudiments and/or joints were recorded. Bone formation was examined in detail in five skeletal elements, namely the scapular blade, humerus, ulna, femur and tibia. Only one distal element of the forelimbs and hindlimbs was measured as the progression of ossification was not observed to be dramatically different between adjacent elements (e.g., radius and ulna, Fig. 1). The pelvis was not examined due to the damage that occasionally occurred when the embryos were divided in half. For each of the five skeletal elements, measurements of the Alcian blue and Alizarin red stained regions were taken at the ventral or dorsal aspect to give the length of the skeletal element and the length of bone in each rudiment, as indicated in Fig. 1. The extent of mineralization in the spine of the scapula was also measured, as indicated between the arrowheads in Fig. 2. For most rudiments, the length was the maximum length parallel to the longitudinal axis of the skeletal element. When a large curvature was present, such as was often found in the tibia, the longitudinal length was recorded as the sum of the lengths of each approximately linear segment. In order to normalize for changes in rudiment length when calculating an effect on calcification, the proportion of each rudiment occupied by bone was then calculated to reflect possible differences in the progression of ossification relative to the length of the rudiment.

### Acquisition and analysis of 3D data

**Fig. 3:**
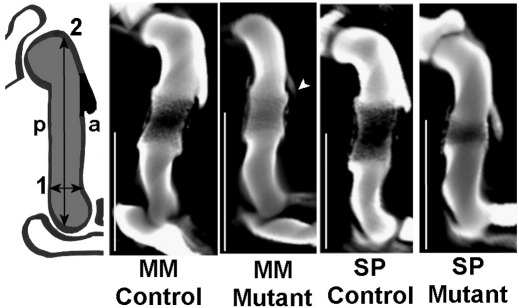
Characteristics of the humerus in control and mutant limbs at TS23. The outline drawing on the left represents the plane of longitudinal section (anterior to posterior aspect; a, p) through 3D computer reconstructions of control and mutant humeri shown. The size of the humeral tuberosity was measured by normalizing the area of the tuberosity (shown in black on the left) to the total area of the humerus (shown in grey). The size of the humeral tuberosity was significantly reduced (*p* < 0.05) in the *Myf5^nlacZ/nlacZ^:Myod^−/−^* (MM) mutants compared to stage-matched controls (arrow). While the size of the *Pax3*^*Sp/Sp*^ (SP) humeral tuberosities was not significantly different to those of controls, the shape of the tuberosity was different (SP Mutant). The tapering of the distal humerus was quantified by normalizing the narrowest width of the distal humerus (1) to the longest parallel length of the humerus (2). It was found that both types of mutants had significantly less (*p* < 0.01) tapering than their littermate controls. Scale bars 1 mm.

Six cartilage-stained limbs at TS23 from each of the two muscleless mutant groups (*Myf5^nlacZ/nlacZ^:Myod^−/−^* and *Pax3*^*Sp/Sp*^) and the same number of control littermates were selected for 3D imaging. These limbs were scanned using optical projection tomography (OPT) [27] to obtain 3D data on morphology and distribution of Alcian blue stained tissue (as further described in [28,29]). The morphology of stained tissue was visualized with visible light and a 750 nm filter. Software (MAPaint) developed and provided by the Edinburgh Mouse Atlas Project [30] was used to navigate through the 3D data and view virtual sections through two joints, the elbow joint in the forelimb and the femoro-tibial (knee) joint in the hindlimb (known as the stifle in quadrupeds). The 3D data were also used to quantify the size of the humeral tuberosity, the tapering of the distal humerus, and the flaring of the proximal tibia and radius. The size of the humeral tuberosity was estimated by normalizing the area of the tuberosity to the total area of the humerus, with the areas measured from virtual longitudinal sections taken at the anterior to posterior aspect from 3D reconstructions of the humerus, as illustrated in Fig. 3. The tapering of the humerus was measured by normalizing the narrowest width (anterior to posterior aspect) of the distal humerus in longitudinal sections to the length of the humerus, as shown in Fig. 3. Flaring of the proximal tibia and radius was also measured from longitudinal sections by calculating the ratio of widths (from anterior to posterior) at the narrowest and widest parts of the proximal tibia and radius, as indicated by dotted lines and numbered 1 and 2 in Fig. 1.

### Histology

Limbs of TS23 *Myf5^nlacZ/nlacZ^:Myod^−/−^*, *Pax3*^*Sp/Sp*^, *Myf5^nlacZ/+^:MyoD^−/−^* (reduced muscle) and control littermates were fixed in 4% paraformaldehyde at 4 °C overnight, dehydrated through an ethanol series and cleared in Histoclear-II (National Diagnostics), embedded in pure paraffin wax (Acros-organics, New Jersey) and sectioned at 10 μm using a Leica RM2255 microtome. The sections were collected on superfrost-plus slides and stained with Weigert's Iron Hematoxylin, Fast green and Safranin-O (WIH/FG/SO). Sections were stained for 30 s in freshly prepared Weigert's Iron Hematoxylin working solution, composed of equal proportions of solution A; 1% Hematoxylin (Fisher Scientific) in 95% ethanol, and solution B; 4 ml 29% Ferric chloride, 1 ml concentrated HCl and 95 ml of water. Sections were then stained with 0.02% solution of Fast Green (Sigma) for 5 min and then in 0.1% solution of Safranin-O (Sigma) for another 5 min.

### Statistical methods

The TS23 data were analyzed in the statistical package R (http://www.r-project.org/), last accessed August 2009), and standard *t*-tests were performed to compare data sets. The normality of the data was verified using Shapiro-Wilk tests, and the variance of each dataset was tested so that the correct *t*-test could be used. A *p*-value of less than 0.05 was taken as a statistically significant difference. The *Myf5^nlacZ/nlacZ^:Myod^−/−^*, *Myf5^nlacZ/+^:MyoD^−/−^* and *Pax3*^*Sp/Sp*^ mutants were compared to the stage-matched controls of the equivalent strain, and *t*-tests were also used to compare these mutants. Due to the small sample size, *t*-tests were not performed on the *Pax3*^*Sp/Sp*^ TS25 data.

## Results

### Gross skeletal morphology in mutant limbs

Several prominent abnormalities were observed in forelimb skeletal rudiments of the *Myf5^nlacZ/nlacZ^:Myod^−/−^* and *Pax3*^*Sp/Sp*^ mutants, as shown in Fig. 1 and enumerated in Table 1. Initiation of ossification was abnormal in all mutant scapulae, with a range of abnormalities in evidence. In two *Pax3*^*Sp/Sp*^ mutants, calcification of the scapular blade had not yet commenced, as shown in Fig. 2C, even though mineralization had initiated in more distal elements (radius and ulna). In 4 out of 10 *Myf5^nlacZ/nlacZ^:Myod^−/−^* mutants, and a further 3 out of 7 TS23 *Pax3*^*Sp/Sp*^ mutants, the calcified region of the scapular blade did not extend across the width of the entire element (Figs. 1C and G). All mutants displayed a mismatch between ossification of the scapular blade and scapular spine, where calcification of the spine appeared more advanced relative to ossification of the blade, as shown in Figs. 2B and C, compared to the scapulae of control embryos in which the blade and spine were calcified to the same, or similar, extent, as shown in Fig. 2A. Quantitative analysis of bone development in both components of the scapula is presented below. In the humerus, while the calcified region in control embryos forms a regular band around the mid-diaphysis, an abnormal pattern of calcification was observed in nineteen out of twenty of the ‘muscleless’ limbs. In these muscleless mutants the humeral bone formed as a non-uniform region at the mid-diaphysis, with more extensive calcification on the posterior side of the rudiment (Figs. 1C, G and K, and 2E and F) while mineralized territories in the radius and ulna appeared normal in shape. In the *Myf5^nlacZ/nlacZ^:Myod^−/−^* mutants, the humeral tuberosity appeared absent, or greatly reduced in size (Fig. 1C), while in the *Pax3*^*Sp/Sp*^ mutants the humeral tuberosity did not appear dramatically decreased in size (Fig. 1K). No gross abnormalities were evident in the hindlimbs of the *Myf5^nlacZ/nlacZ^:Myod^−/−^* and *Pax3*^*Sp/Sp*^ mutants (Fig. 1). In *Myf5^nlacZ/+^:MyoD^−/−^* embryos, which have reduced muscle mass, no abnormalities in ossification site morphology were observed.

### Shape changes in skeletal rudiments in muscleless limbs

The size of the humeral tuberosity was quantified by measuring the area from longitudinal sections through the anterior–posterior axis. 3D computer reconstructions of scanned Alcian blue stained limbs permitted virtual sections to be taken in the same orientation through 6 *Myf5^nlacZ/nlacZ^:Myod^−/−^*, 6 *Pax3*^*Sp/Sp*^ mutants and 6 controls for each group (all TS23). The area of the humeral tuberosity in each case was normalized to the area of the humerus in the same section. It was found that the size of the humeral tuberosity was significantly reduced in the *Myf5^nlacZ/nlacZ^:Myod^−/−^* mutants compared to control littermates (*p* < 0.05), as illustrated in Fig. 3. No significant difference was found in the area of the humeral tuberosity between *Pax3*^*Sp/Sp*^ mutants and controls, but a difference in shape was evident in all *Pax3*^*Sp/Sp*^ mutants, whose humeral tuberosities tended to be longer, thinner and less attached to the humerus than control littermates, as shown in Fig. 3.

Shape differences were also noted in the thickness and tapering of the distal humerus and proximal radius where they interface with the elbow joint. The tapering of the distal humerus was characterized by normalizing the width in the anterior to posterior orientation at the narrowest part of the distal humerus to the length of the humerus, as illustrated in Fig. 3. It was found that there was significantly less tapering (*p* < 0.01) of the distal humerus in the *Myf5^nlacZ/nlacZ^:Myod^−/−^* and *Pax3*^*Sp/Sp*^ TS23 mutants than in control littermates, with the mutant humeri exhibiting a more regular width at the distal end than the tapering distal end of the control humeri, as shown in Fig. 3. The flaring of the proximal tibia and radius was also measured, and it was found that both mutants at TS23 exhibited significantly less (*p* < 0.01) flaring at the proximal end of the radius than measured in the controls, while no significant differences between mutants and controls were found in the flaring of the proximal tibia.

### Abnormal joint formation in muscleless limbs

**Fig. 4:**
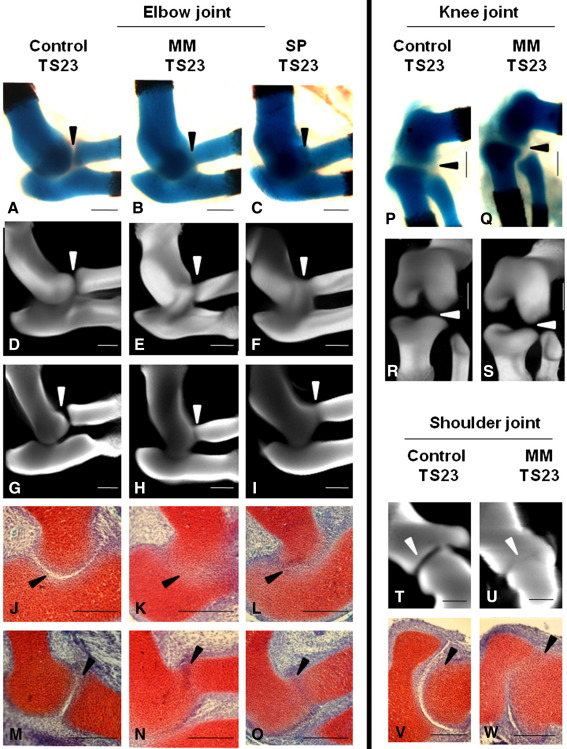
Joint line reduction in limbs devoid of skeletal muscle in *Myf5^nlacZ/nlacZ^:Myod^−/−^* (MM) and *Pax3*^*Sp/Sp*^ (SP) mutants. The joint line of the elbow is visibly reduced in the mutant limbs as shown by photographs of Alcian blue stained cartilage rudiments (A–C), external views of 3D reconstructions of the Alcian blue stained joints (D–F), virtual sections through the 3D reconstructions (G–I), and by histological sections showing the humerus–ulna joint (J–L) and the humerus–radius joint (M–O). The joint line of the knee is not visibly reduced, as shown by photographs (P–Q) and external views of 3D reconstructions of Alcian blue stained tissue (R–S). The scapula–humerus joint line of the shoulder is reduced, shown in sections of 3D reconstructions Alcian blue stained tissue (T–U) and by histological sections (V–W). Histological sections stained with Weigert's Iron Hematoxylin/Fast green/Safranin-O. Scale bars 200 μm.

The joint regions were differentially affected in the limbs of mice devoid of muscle. In TS23-staged *Myf5^nlacZ/nlacZ^:Myod^−/−^* (MM) and *Pax3*^*Sp/Sp*^ (SP) mice, the elbow was the most severely affected, with a reduction in the joint line of the elbow evident in superficial photographs (Figs. 4A–C) whereas the knee joint appeared unaffected (Figs. 4P, Q). The abnormalities in the elbow joint were examined in more detail using 3D reconstructions of Alcian blue stained forelimbs and histological sections. External views of the reconstructed rudiments show a dramatic reduction in the sharp definition of the rudiment territories and the separation of the rudiments in the elbow (Figs. 4D–F), whereas no change in definition of the boundaries was seen in mutant knee joints (Figs. 4R–S). Virtual sections through the 3D reconstructions of the elbow confirm the loss of sharp definition in the Alcian blue stained tissue (Figs. 4G–I). Histological sections, stained with Safranin-O, showed a complete loss of cellular organization of the interzone between the cartilage rudiments of the elbow in both *Myf5^nlacZ/nlacZ^:Myod^−/−^* (MM) and *Pax3*^*Sp/Sp*^ (SP) mutants (Figs. 4J–O). Normal separation between the rudiments of the elbow joint was seen in the reduced muscle (*Myf5^nlacZ/+^:MyoD^−/−^*) mutants (data not shown). The shoulder joint also showed a reduction in the joint line, and a similar alteration of tissue organization as seen in the elbow was found in the scapula–humerus interface of the shoulder joint (Figs. 4T–W).

### Quantitative analysis of the extent of bone initiation and progression in muscleless limbs

**Fig. 5:**
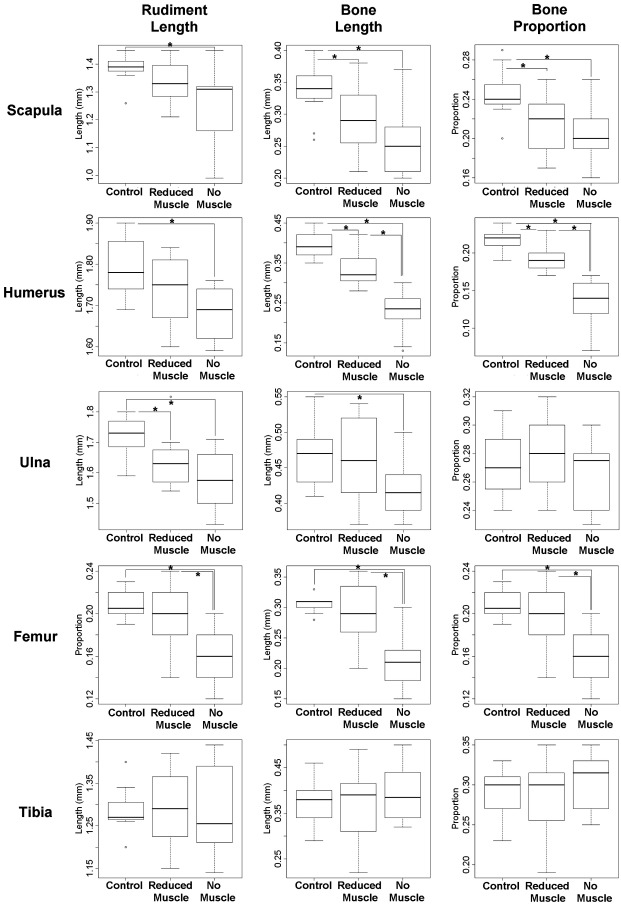
Comparison of ‘Reduced Muscle’ phenotype (*Myf5^nlacZ/+^:Myod^−/−^*), ‘No Muscle Phenotype’ (*Myf5^nlacZ/nlacZ^:Myod^−/−^*) and control littermates in which skeletal muscle develops normally. Statistical significant differences (*t*-test, *p* < 0.05) between groups are indicated by an asterisk (⁎). Rudiment length, length of bone and proportion of rudiment composed of bone are shown for the scapula, humerus, ulna, femur and tibia. Specimen numbers are given in Table 2.

**Fig. 6:**
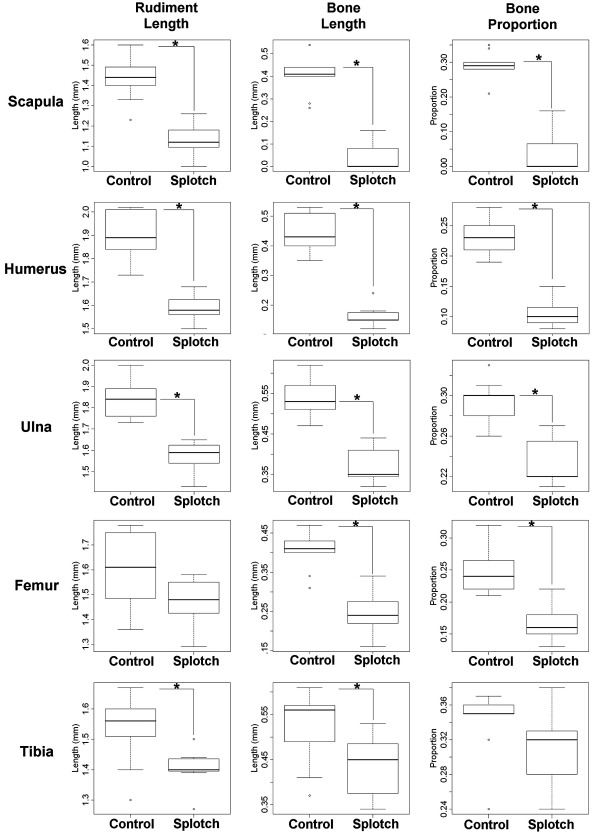
Means and standard deviations of *Pax3*^*sp/sp*^ (splotch) at TS23 and TS25 with stage-matched control littermates. *t*-tests were performed for TS23 data only, as TS25 dataset size was too small. Statistical significant differences (*t*-test, *p* < 0.05) between TS23 mutants and controls are indicated by an asterisk (⁎). Specimen numbers are given in Table 2.

To compare the early stages of ossification in muscleless mutant limbs and control littermates with normal muscle mass, three parameters were recorded for the scapular blade, humerus, ulna, femur and tibia of each specimen; these parameters were the longitudinal length of the rudiment (rudiment length), the longitudinal length of calcified tissue in the rudiment (bone length), and the proportion of the rudiment composed of calcified tissue (bone proportion) (Table 2, Figs. 5 and 6). Significant differences were found for all three parameters in the scapula, humerus and femur, and for ulnar length and bone length in the *Myf5^nlacZ/nlacZ^:Myod^−/−^* TS23 mutants, while ulnar bone proportion and all three measurements of the tibia showed no significant differences, as detailed in Table 2, and illustrated in Fig. 5. The extent of calcification of the scapular spine was also measured, and it was found that there was no significant difference between the *Myf5^nlacZ/nlacZ^:Myod^−/−^* mutants and controls in the amount of bone in this region of the scapula (not shown). Therefore the relative advancement of ossification in the spine compared to the blade of the scapula noted from Fig. 1 is due to a reduction in ossification in the blade, and is not due to an increase in bone formation in the scapular spine. There were significant differences between the *Myf5^nlacZ/nlacZ^:Myod^−/−^* (muscleless) mutants and the *Myf5^nlacZ/+^:Myod^−/−^* (reduced muscle) mutants for the amount of humeral bone present (bone length and proportion), and for all three measurements of the femur, while the scapula, humerus and ulna showed no significant difference between the muscleless and reduced muscle phenotypes (Table 2 and Fig. 5). When the reduced muscle phenotype was compared with control littermates, significant differences were found for ulnar length, scapular (blade) bone and humeral bone, while all other parameters and rudiments showed no significant differences to the control littermates (Table 2, Fig. 5). Like the *Myf5^nlacZ/nlacZ^:Myod^−/−^* mutants, the *Pax3*^*Sp/Sp*^ TS23 mutants showed significant decreases in all three parameters in the scapula and humerus, and no significant difference in ulnar bone or the proportion of bone in the tibia (Fig. 6). In contrast to the *Myf5^nlacZ/nlacZ^:Myod^−/−^* mutants, the *Pax3*^*Sp/Sp*^ TS23 mutants showed no significant change in femoral length, and did show a significant difference in the length and bone length of the tibia, and the proportion of bone in the ulna (Fig. 6). The *Pax3*^*Sp/Sp*^ TS23 mutants also had significantly less ossification of the scapular spine in comparison to controls (*p* < 0.05). Due to the small sample size of the *Pax3*^*Sp/Sp*^ TS25 mutants (*n* = 3), *t*-tests were not performed. However, the measurements (as detailed in Table 2) indicate that some of the effects seen earlier in development are still in evidence at this time point with reduced rudiment length and impeded bone formation in the scapula, humerus, ulna and femur.

**Fig. 7:**
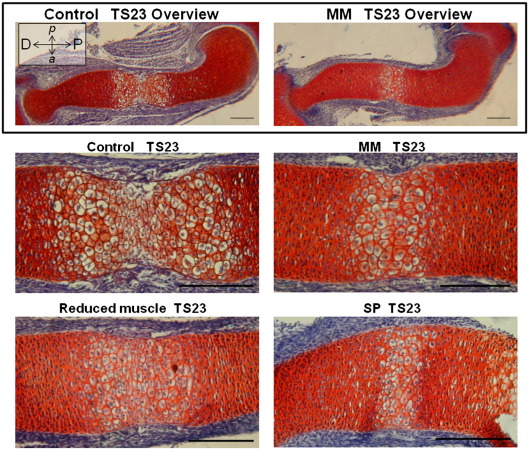
Histological analysis of primary ossification centers in TS23 *Myf5^nlacZ/nlacZ^:Myod^−/−^* (MM), *Myf5^nlacZ/+^:Myod^−/−^* (reduced muscle), *Pax3*^*Sp/Sp*^ (SP) and control humeri. Orientation of sections is as indicated in top left corner (D; distal, P; proximal, *a*; anterior, *p*; posterior). The zone of hypertrophic chondrocytes in the mid-diaphysis of the humerus is reduced in limbs devoid of muscle (*Myf5^nlacZ/nlacZ^:Myod^−/−^* (MM) and *Pax3*^*Sp/Sp*^ (SP) mutants) compared to stage-matched controls. *Myf5^nlacZ/+^:Myod^−/−^* embryos with reduced muscle have a hypertrophic zone that is intermediate in extent between the control and muscleless limbs. Sections stained with Weigert's Iron Hematoxylin/Fast green/Safranin-O. Scale bars 200 μm.

As the humerus was one of the most severely affected rudiments, histological analysis was performed to analyze the primary ossification center (POC) of TS23 muscleless (*Myf5^nlacZ/nlacZ^:Myod^−/−^* and *Pax3*^*Sp/Sp*^), reduced muscle (*Myf5^nlacZ/+^:Myod^−/−^*) and control humeri. Histology of control humeri revealed two fronts of hypertrophic chondrocytes, with matrix degradation indicative of bone deposition at the mid-diaphysis (Fig. 7). In contrast, a single, more uniform region of hypertrophic chondrocytes was detectable in the *Myf5^nlacZ/nlacZ^:Myod^−/−^* and *Pax3*^*Sp/Sp*^ mutants, and the extent of the entire POC in these mutants was much decreased in comparison to those of the control limbs, as shown in Fig. 7. The absence of evidence of matrix degradation in the POC in the mutant limbs indicates that substantial bone deposition had not yet begun. The extent of the POC in reduced muscle mice (*Myf5^nlacZ/+^:Myod^−/−^*) was intermediate to those of the controls and muscleless mutants, and the mid-diaphyseal region was more uniform and homogeneous than seen in control limbs (Fig. 7). Interestingly, the wedge-shaped pattern of mineralization seen in intact mutant humeri (Figs. 1 and 2) was not in evidence in the shape of the hypertrophic zones, which were uniform along the anterior–posterior axis. In contrast to what was seen in the humerus, histological analyses revealed no differences between mutant and control tibiae (not shown).

## Discussion

In this study, skeletal development was characterized in mouse embryo mutant limbs devoid of skeletal muscle. Both types of muscleless mutants examined (*Myf5^nlacZ/nlacZ^:Myod^−/−^* and *Pax3*^*Sp/Sp*^) showed differences in the size and shape of rudiments, the pattern of onset of ossification sites within specific rudiments and the separation of rudiments at specific joints. A quantitative analysis of ossification in a number of rudiments revealed significantly less bone in the scapular blade, humerus, ulna and femur, but no significant change in the progression of ossification in the tibia in both muscleless mutants. The morphology of ossification centers was affected in the scapula and humerus, with non-uniform or absent calcified regions in the scapulae of mutants, and non-uniform morphology of bone in the humerus. Histological analysis of the primary ossification center in the humerus revealed a reduction in the size of the hypertrophic region and impeded progression of the growth plates. In both muscleless mouse models, ossification sites were differentially affected, with a trend indicating that bone formation may be more severely affected in the proximal elements than in the distal elements (scapula and humerus vs. ulna), and that calcification of the major long bones in the forelimb may be more dependant on muscle contractions than in the hindlimb elements (e.g., ulna vs. tibia). Joint development and rudiment shape also showed differential effects of the absent musculature. The elbow joint was affected by the lack of skeletal muscle, with a dramatic reduction in the definition of the rudiment territories and the separation of the rudiments, whereas no change in definition or separation was seen in mutant knee (stifle) joints. The shape of several elements was affected, with the mutants exhibiting less tapering of the distal humerus and less flaring of the proximal radius, but unchanged flaring of the proximal tibia. The size of the humeral tuberosity was found to be reduced in all of the *Myf5^nlacZ/nlacZ^:Myod^−/−^* mutants, and while the size of the tuberosity was unaffected in the *Pax3*^*Sp/Sp*^ mutants, the shape was altered by comparison with controls. A mutant in which limb musculature is reduced but not absent was also examined (*Myf5^nlacZ/+^:Myod^−/−^*). The ‘reduced muscle’ phenotype produced an intermediate effect on the limbs, with a shorter ulna and significantly less scapular and humeral bone than in control littermates (as also seen in the muscleless *Myf5^nlacZ/nlacZ^:Myod^−/−^* limbs), but with all other measurements of the reduced muscle phenotype showing no significant differences to controls.

We have described how the lack of skeletal muscle affects skeletal development at TS23, the developmental stage at which the ossification centers of the major long bones form. Some of the findings at TS23 correlate with later developmental stages, such as the shorter scapula and femur reported by Rot-Nikcevic et al. [17] and the shorter ulna described by Gomez et al. [19]. Previous studies [17,19] reported that the lengths of different rudiments were not equally affected by the lack of muscle in later stage embryos, and we show similar findings at TS23; such as significant reduction in length of the scapula, humerus, ulna and femur but not of the tibia in the *Myf5^nlacZ/nlacZ^:Myod^−/−^* mutants. Gomez et al. [19] reported thicker humeri and femora in E18 muscleless mutants, while at TS23, we found that the lack of muscle had a differential effect on the shape of the rudiments, with the distal humerus and proximal ulna significantly affected but with no significant difference in the proximal tibia. In contrast to the findings of previous studies [17,19] that the humeral tuberosity is absent in muscleless mice at E18–18.5, our results demonstrate that the humeral tuberosity is present at TS23, albeit reduced in size in the *Myf5^nlacZ/nlacZ^:Myod^−/−^* mutants. This indicates that initiation of the humeral tuberosity occurs, but maintenance and outgrowth of the tuberosity might be dependent on mechanical stimulation from the muscle in the *Myf5^nlacZ/nlacZ^:Myod^−/−^* mutants. The shape but not size of the humeral tuberosity was altered in the *Pax3*^*Sp/Sp*^ mutants, suggesting that the humeral tuberosity may be impacted by the presence of muscle progenitor cells in the *Myf5^nlacZ/nlacZ^:Myod^−/−^* mutants, or by specific outcomes of the different genetic lesions.

We show that the initiation and progression of long bone ossification centers are differentially affected by the absence of skeletal muscle with abnormal and reduced bone formation in several, but not in all, rudiments. Gomez et al. [19] reported that ossification is equally developed and structured in the digits of mutant and control embryos at E18. As ossification in the digits does not start until TS25, we did not analyze the progression of bone formation in these rudiments in our animals. It is possible that ossification of the digits is unaffected by the lack of skeletal muscle, as was found for the tibia in our study, or alternatively, that differences in bone development similar to those we have observed are no longer apparent by the later stage of E18.

The results obtained in this study demonstrate that developing rudiments are differentially affected by the absence of skeletal muscle in the limbs, for cartilage growth, bone formation and joint development, indicating a complex relationship between skeletogenesis and the biophysical environment. The comparative study of several rudiments shows an intriguing correlation between severity of effect and position of the rudiment on the anteroposterior axis (forelimb vs. hindlimb) and proximodistal axis. The scapula (blade) and humerus were severely affected in the muscleless mutants, with alterations in morphology of ossification centers, and changes in the lengths of the cartilage rudiments and the calcified regions. The scapular blade and humeral bone regions seem to be highly dependent on certain levels of biophysical stimuli induced by muscle contractions, as their ossification is affected even when a reduced amount of muscle is present, while other rudiments are not significantly affected. In the *Myf5^nlacZ/nlacZ^:Myod^−/−^* mutants, while the blade of the scapula was significantly affected, the scapular spine was not. Intriguingly, it seems that the effect of absent muscle may depend on embryonic origin of the cells, as the scapula has a dual embryonic origin; the spine being somatopleure and the blade being of somite origin [31]. Joint formation was also differentially affected, as histological analysis showed how the cellular organization was almost completely lost in the mutant elbow joint, and 3D data revealed a clear joint line in the mutant knee (stifle), but not in the elbow.

The differential effects of skeletal muscle on skeletogenesis described in this study could be due to altered mechanical forces, or to missing trophic factors from the muscle bodies, or to a combination of these influences. However, the intermediate effects seen in the ‘reduced muscle’ phenotype (*Myf5^nlacZ/+^:Myod^−/−^*), indicate that the effects on the mutant limbs are unlikely to be from trophic factors from the muscle alone. The fact that aspects of the ‘reduced muscle’ phenotype were normal, such as joint formation and hindlimb ossification, may imply that a minimum threshold of mechanical forces must be reached for normal skeletal development. It is possible that forces due to muscle contractions are supplemented by external mechanical stimulation, such as the forces incurred by uterine contractions, or by the movement of the mother and littermates in the uterus. Certain regions of the developing limb may be subject to higher ‘external’ forces, and the biophysical stimuli induced by these forces could be high enough to compensate (at least in part) for the lack of skeletal muscle in elements such as the tibia. Planned finite element analyses will characterize levels of biophysical stimuli in the various skeletal elements for control and reduced muscle limbs, and may offer clues as to how the altered mechanical environment can yield such a range of effects on bone formation. Alternatively, it is possible that the minimum ‘threshold’ of mechanical forces varies depending upon location in the embryonic limb, meaning that some parts of the developing limb are more sensitive to mechanical stimulation than others due to the molecular context in that region. It has been shown that certain regulatory genes are expressed differently in the early developing forelimb and hindlimb, with a survey of Wnt gene expression patterns in the limb bud showing differences in transcript distribution between forelimb and hindlimb [29] and a genome wide analysis of transcripts differentially expressed in the fore and hind limbs demonstrating differences in the use of Fgf, Hedgehog and BMP family signalling molecules [32]. Therefore, variation in the molecular context of developing skeletal rudiments in different parts of the developing fore and hind limbs may influence the impact of the altered mechanical environment resulting from absence of muscles.

In conclusion, skeletal development is differentially affected by the lack of skeletal muscle, with certain rudiments, ossification sites and joints being more severely affected than others. These findings indicate a complex interaction between mechanical forces and location-specific regulatory factors impacting on bone and joint development. The results have implications for tissue engineering of bone and articular cartilage, as they imply that mesenchymal cells in the embryonic limb may respond differentially to biophysical stimuli, depending upon their location and environment.

## Figures and Tables

**Table 1:** Frequency of occurrence of the most prominent skeletal abnormalities noted in *Myf5^nlacZ/nlacZ^:Myod^−/−^* (MM) and *Pax3*^*Sp/Sp*^ (SP) mutants.

	Total number of mutants	Abnormal elbow joint	Abnormal scapular bone	Abnormal humeral bone	Reduced humeral tuberosity
MM, TS23	10	10	10	9	10
SP, TS23	7	7	7	7	0
SP, TS25	3	3	3	3	0

**Table 2:**
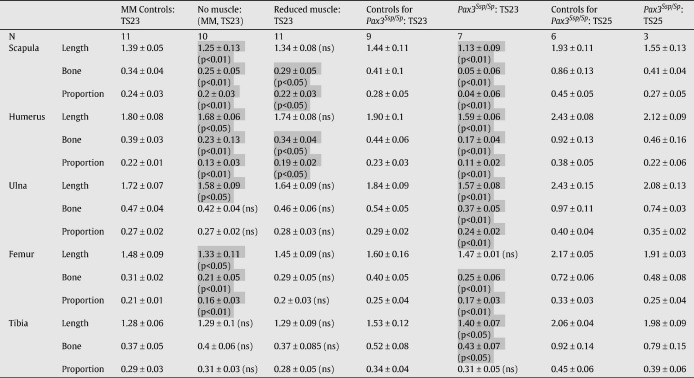
Mean and standard deviations for rudiment length, bone length and proportion of bone length to rudiment length in ‘muscleless’ *Myf5^nlacZ/nlacZ^:Myod^−/−^* (MM) mutants, reduced muscle mutants (*Myf5^+/nlacZ^:Myod^-/^*) and control littermates (MM Controls) at TS23, and ‘muscleless’ splotch (*Pax3*^*Sp/Sp*^) mutants and control littermates at TS23 and TS25. *p*-values shown indicate a statistically significant difference in the TS23 mutants relative to the control littermates. *t*-tests were not performed on TS25 data due to the small sample size. Significant differences between mutant and control littermate groups are highlighted in gray.
